# Effect of counter-pulsation control of a pulsatile left ventricular assist device on working load variations of the native heart

**DOI:** 10.1186/1475-925X-13-35

**Published:** 2014-04-03

**Authors:** Seong Wook Choi, Kyoung Won Nam, Ki Moo Lim, Eun Bo Shim, Yong Soon Won, Heung Myong Woo, Ho Hyun Kwak, Mi Ryoung Noh, In Young Kim, Sung Min Park

**Affiliations:** 1 Department of Mechanical and Biomedical Engineering, Kangwon National University, Chuncheon, Korea; 2 Department of Biomedical Engineering, Hanyang University, 222 Wangsimni-ro, Seongdong-gu, Seoul 133-791, Korea; 3 Department of Medical IT Convergence Engineering, Kumoh National Institute of Technology, Gumi, Korea; 4 Cardiac Surgical Clinic, Bucheon, Korea; 5 Stem Cell Institute, College of Veterinary Medicine, Kangwon National University, Chuncheon, Korea; 6 School of Medicine, Kangwon National University, Chuncheon, Korea

**Keywords:** Left ventricular assist device, Counter-pulsation control, Left ventricle working load, Ventricular electrocardiogram

## Abstract

**Background:**

When using a pulsatile left ventricular assist device (LVAD), it is important to reduce the cardiac load variations of the native heart because severe cardiac load variations can induce ventricular arrhythmia. In this study, we investigated the effect of counter-pulsation control of the LVAD on the reduction of cardiac load variation.

**Methods:**

A ventricular electrocardiogram-based counter-pulsation control algorithm for a LVAD was implemented, and the effects of counter-pulsation control of the LVAD on the reduction of the working load variations of the left ventricle were determined in three animal experiments.

**Results:**

Deviations of the working load of the left ventricle were reduced by 51.3%, 67.9%, and 71.5% in each case, and the beat-to-beat variation rates in the working load were reduced by 84.8%, 82.7%, and 88.2% in each ease after counter-pulsation control. There were 3 to 12 premature ventricle contractions (PVCs) before counter-pulsation control, but no PVCs were observed during counter-pulsation control.

**Conclusions:**

Counter-pulsation control of the pulsatile LVAD can reduce severe cardiac load variations, but the average working load is not markedly affected by application of counter-pulsation control because it is also influenced by temporary cardiac outflow variations. We believe that counter-pulsation control of the LVAD can improve the long-term safety of heart failure patients equipped with LVADs.

## Background

When a pulsatile left ventricular assist device (LVAD) is used to support the blood pumping function of the damaged native heart of a patient in end-stage heart failure, it is important to consistently supply sufficient blood flow to the aorta [1-3]. Furthermore, it is important to reduce the variations in the physical load of the left ventricle (LV) during LVAD support because severe cardiac load variations can shorten the monophasic action potential duration of repolarization of the native heart (QT interval), thus increasing the risk of ventricular tachycardia or fibrillation [4-9]. Most pulsatile LVAD systems are connected to the LV and the aorta in parallel, allowing for simultaneous blood pumping by the native heart and the LVAD without any ejection-timing control of the LVAD, which can generate temporary severe cardiac load variations that can threaten long-term patient safety. To reduce the risk of LVAD support, the incidence of these temporary severe cardiac load variations must be reduced. This can be realized by counter-pulsating control of the LVAD, which prevents the simultaneous co-pulsation of the native heart and the LVAD [10].

Several studies have investigated the relationship between the control scheme of the LVAD and the working load of the native heart. For example, Shi *et al*. assessed the hemodynamic benefits of three pumping modes (constant, counter-pulsation, and co-pulsation) using a diseased cardiovascular model and three VAD models (positive displacement, impeller, and a novel reciprocating-valve design) [11]. In their study, the working load of the LV was lower using the displacement pump and the reciprocating-valve pump in counter-pulsation mode. In addition, Lim *et al*. implemented a deadbeat controller that could operate an implantable rotary blood pump in a pulsatile manner using pump speed and driver current measurements [12]. In their simulation using *in vivo* porcine model data and *in vitro* mock loop data, counter-pulsation produced minimal stroke work and LV end-diastolic volume compared with other control schemes. These simulation studies demonstrated the benefits of counter-pulsation control of the LVAD, but neither performed animal experiments to identify the actual effects of counter-pulsation control on the working load of the native heart. Therefore, although these previous simulation studies mathematically proved the benefit of counter-pulsation control of the LVAD, it is also worth observing the actual effects of counter-pulsation control on the working load of the native heart using animal models.

In this study, the clinical effects of counter-pulsation control of the pulsatile LVAD on the reduction in working load variations of the LV during LVAD support were evaluated in an animal model.

## Methods

### Brief description of the pneumatic LVAD used

**Figure 1:**
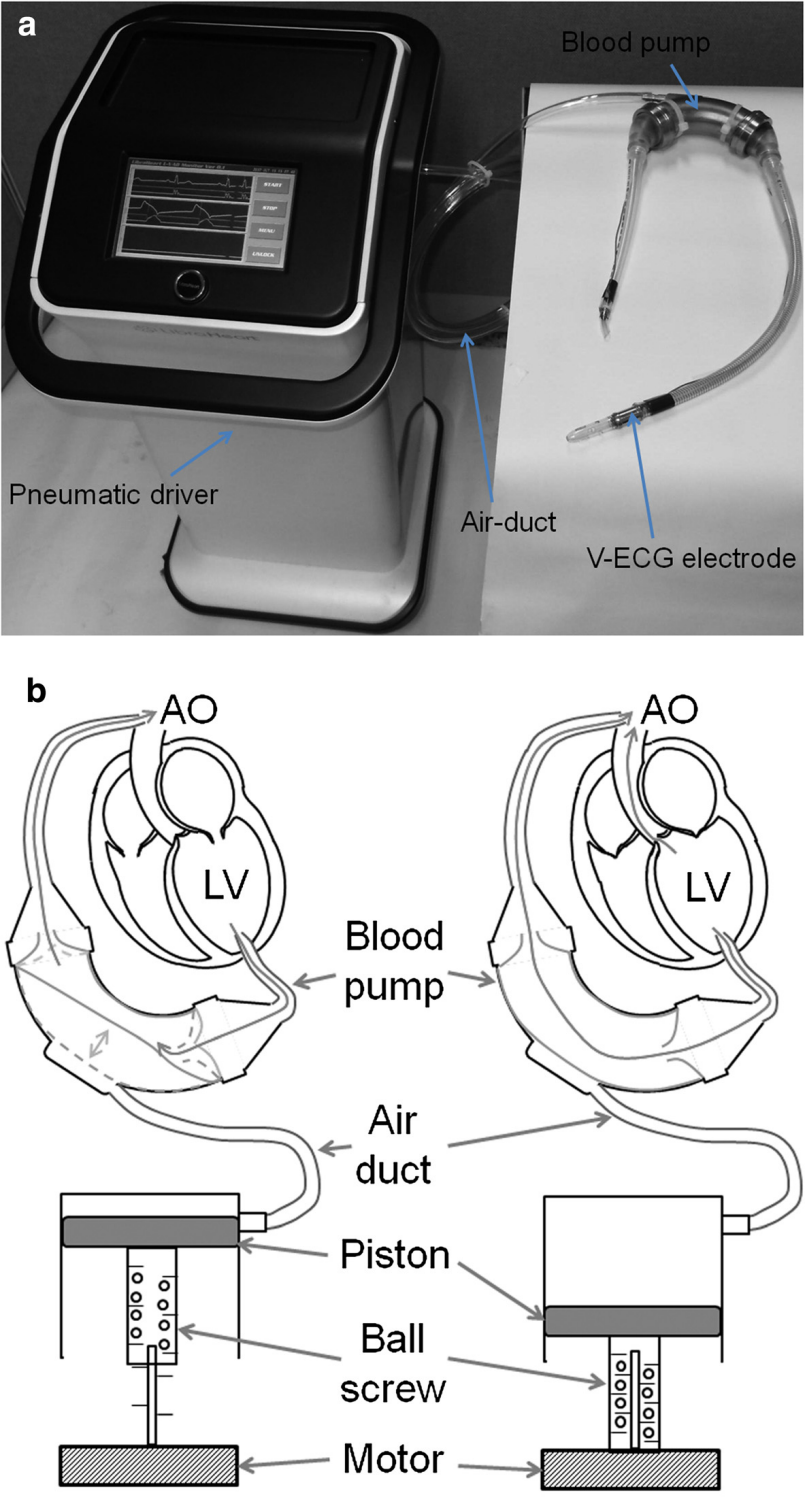
LibraHeart I pulsatile LVAD system (a) and its operating mechanism under normal situations (left) and in the power-off situation (right) (b).

This study utilized a portable pneumatic LVAD, the LibraHeart I, which is under development by the Kangwon National University and LibraHeart Inc. (Chuncheon, Korea) [13-15]. It comprises three sub-parts: 1) a portable pneumatic driver comprising a brushless DC motor, a ball screw, a piston, and a cylinder; 2) an implantable blood pump comprising a diaphragm, inlet and outlet cannulae, two one-way valves, and two unipolar leads and platinum plates on the surface of the inlet and output cannulae; and 3) an air duct with two solenoid one-way air valves (Figure 1a). It was designed such that the maximal outflow is ≥ 5 L/min, the one-stroke volume is 50 mL, and the maximal pumping rate is 120 bpm. The implantable blood pump was designed to be a bent-tube shape to reduce its own flow resistance. The inlet cannula of the blood pump is connected to the LV apex of the native heart, and the outlet cannula of the blood pump is connected to the aorta. When the pneumatic driver begins operating, an outlet air valve on the air duct is closed, and the pneumatic pressure in the air duct is then manually adjusted via an inlet air valve on the air duct so that the blood pump operates in full-filling and full-ejection states. Figure 1b demonstrates the operating mechanism of the LibraHeart I under normal situations (left) and in the power-off situation (right). In normal situations, the LibraHeart I plays the role of a pulsatile LVAD that assists the blood-pumping function of the damaged native heart: solid lines in the blood pump (diaphragm and valve) represent blood ejection and dashed lines represent blood-filling periods of the LVAD, respectively. When the operation of the pneumatic driver is stopped due to an unpredicted system malfunction or other emergency situation during long-term continuous operation, if the user/operator turns off the system immediately, the outlet air valve on the air duct is opened and pneumatic pressure in the air duct decreases (power-off situation). The blood sac is then maximally enlarged by the LV ejecting force; that is, the blood pump serves as a hollow bent tube and plays the role of a secondary blood path between the LV and the aorta. Thus, the contraction force of the LV can maintain a certain volume of blood flow through the pump, even after the pneumatic driver has stopped if both cannulae are short. In this way, thrombus formation in the blood sac is delayed, and if the operator replaces the pneumatic driver and begins re-operation within a short time interval, the damage to the patient can be reduced.

### Implementation of counter-pulsation control

**Figure 2:**
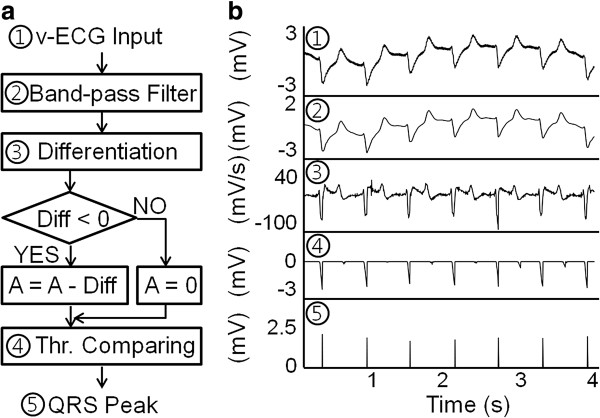
**v-ECG-based QRS peak detection algorithm implemented for LibraHeart I (a) and signal patterns before, during, and after peak detection processing (b).** Thr: threshold.

To measure the ventricular electrocardiogram (v-ECG) signals in a real-time manner using two unipolar leads, platinum plates were fixed onto the surface of the inlet and outlet cannulae of the implantable blood pump [16]. These plates were connected to the controller board in the pneumatic driver console through wire connections. During implantation, the tip of the measuring electrode was fixed to the LV apex, and the tip of the reference electrode was fixed to the ascending aorta by suturing. The measured v-ECG signal was converted into QRS peak signals, which represent the timing of LV contraction, using the following signal processing techniques: band-pass filtering, differentiation, integration, and threshold comparing (Figure 2) [17,18]. More specifically, first, the measured v-ECG signal passed a band-pass filter (lower cut-off frequency = 0.1 Hz and higher cut-off frequency = 50 Hz) to eliminate offset and high-frequency noise components; second, the filtered signal was differentiated and the signal part with negative differential value (Diff in Figure 2a) was extracted from the filtered v-ECG signal; third, the value of the accumulator (A in Figure 2a) was increased by Diff when the sign of the Diff was negative or was cleared to zero when the sign of the Diff was not negative; and fourth, when the value of the accumulator exceeds a preset threshold value (1 mV), the implemented algorithm determines the wave as a QRS peak. Finally, using this information, the ejection timing of the LibraHeart I was adjusted to prevent co-pulsation between the native heart and the LibraHeart I. Table 1 shows the accuracy of the implemented QRS peak detection algorithm based on the v-ECG signal. It showed high (>95%) accuracy in all experiments.

**Table 1:** Accuracy of the implemented v-ECG-based QRS peak detection algorithm during animal experiments

**Animal (dur.)**	**Occurred QRS peak**	**Missed QRS peak**	**Accuracy**
1 (40 min)	3,957	39	99.0%
2 (60 min)	4,501	75	98.3%
3 (25 min)	1,826	61	96.7%

*dur* time duration of peak number counting.

### Animal experiments

Animal experiments using three pigs weighing 35.2 to 40.1 kg were performed with the approval of the Institutional Review Board of Kangwon National University and in compliance with the Ethics and Regulations for Animal Study. The animals were laid on the operating table in a supine position, and 10 mg/kg of intramuscular ketamine was injected to induce anesthesia. Anesthesia was maintained during the surgical procedure and the sensor signal-monitoring period after device operation by consistently supplying 1 – 2% inhaled isoflurane through an endotracheal tube. The inlet cannula was connected to the LV apex, and the outlet cannula was connected to the ascending aorta through a median sternotomy. Table 2 shows the animal characteristics before and during application of the LVAD. In all experiments, the animals survived for 3 h after starting the LVAD operation, and then were euthanized by an anesthetic overdose. Three additional electrodes were attached to both forelegs and the left rear legs of the animals to compare the v-ECG measurements with those of conventional ECG, and the arterial blood pressure (ABP) was measured at the carotid artery. To calculate the working loads of the LV and the LVAD before and after application of the counter-pulsation control, cardiac outflow and LVAD outflow were monitored using two ultrasonic flowmeters (T109 for LVAD and T106 for LV; Transonic Systems Inc., NY, USA; the manufacturer guaranteed error rates under 10% at 36.7° blood condition for both devices). The probe for cardiac outflow measurement was attached to the aortic arch, and the probe for LVAD outflow measurement was attached to the outlet cannula. The working loads (WLs) of the animal’s LV and LVAD were calculated using Eq. (1):

(1) $$\mathrm{WL}\;\left(\mathrm{Joule}/\mathrm{beat}\right)=\int_{t_{0}}^{t_{1}}\left(F\left(t\right)\times P\left(t\right)\right)\mathrm{dt}$$

where t_0_ represents the starting time of heart contraction and t_1_ the ending time of heart contraction. F(t) represents the blood flow through the aortic valve when calculating the LV load and the blood flow through the outlet valve of the LVAD when calculating the LVAD load, respectively. P(t) commonly represents the aortic pressure of the animal in both cases.

**Table 2:** Animal characteristics before and during application of the LVAD

**Animal**	**Weight (kg)**	**Before LVAD application**		**During LVAD application**	
		**ABP (S/M/D) (mmHg)**	**Mean HR (bpm)**	**ABP (S/M/D) (mmHg)**	**Mean HR (bpm)**
1	37.4	65/47/38	84.1	55/46/41	90.1
2	40.1	54/37/30	73.8	48/42/39	89.0
3	35.2	87/59/46	102.3	90/58/40	97.7

*ABP* arterial blood pressure, *S* systole, *M* mean, *D* diastole, *HR* heart rate.

In this study, we substituted the aortic pressure in Eq. (1) with the ABP measurements at the carotid artery because measuring the aortic pressure directly during the experiments was difficult. Carotid artery is placed near the aortic valve and is also directly connected to the ascending aortic arch and therefore, the pressure in the carotid artery is very similar to the aortic pressure. In addition, to observe the variation in the working load of the LV and LVAD during blood pumping before and after application of counter-pulsation control, the beat-to-beat variation rate in the working load (load variation rate, LVR) was calculated using Eq. (2):

(2) $$\mathrm{LVR}\;\left(\%\right)=100\times \frac{\left|{\mathrm{WL}}_{\mathrm{previous}}-{\mathrm{WL}}_{\mathrm{present}}\right|}{{\mathrm{WL}}_{\mathrm{present}}}$$

where WL_previous_ represents the previously calculated working load and WL_present_ represents the present calculated working load, respectively.

## Results

**Figure 3:**
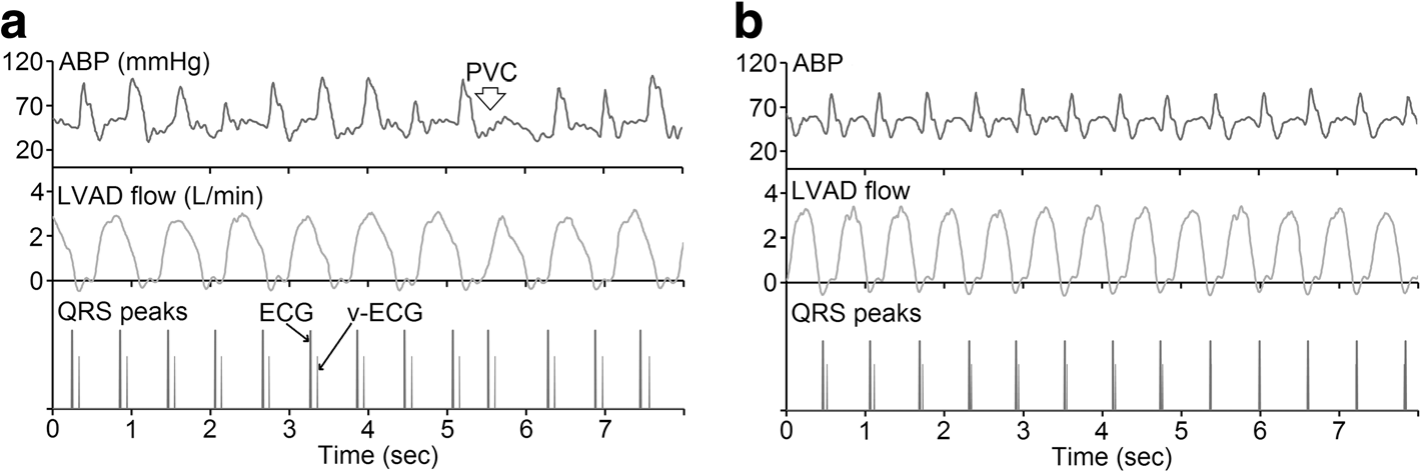
ABP, LVAD outflow, and QRS peaks of v-ECG and ECG signals before (a) and during (b) counter-pulsation control.

During the experiments, the total aortic outflow of the animals was maintained in the range 3.3 – 3.7 L/min, and the heart rate of the animals did not vary substantially before and after LVAD implantation because the animals were under anesthesia. The cardiac outflow was reduced from 3.3 – 3.7 to 1.8 – 2.3 L/min after LVAD implantation; therefore, the pumping rate of the LVAD was set to 70 bpm before counter-pulsation control to regulate the LVAD outflow in the range 1.4 – 1.7 L/min, and was adjusted to 100 bpm to regulate the LVAD outflow in the range 1.5 – 1.9 L/min during counter-pulsation control to maintain the total aortic flow at 3.3 – 3.7 L/min during LVAD support. Figure 3 shows the ABP, outflow of the LVAD, and QRS peaks of v-ECG and ECG signals before and during counter-pulsation control in the first experiment. As seen in Figure 3, co-pulsation between the native heart and the LVAD was successfully prevented and the LVAD outflow pattern was better stabilized after counter-pulsation control (the variation between each ABP pulse signal was reduced, especially at the fourth, eighth, tenth and tenth pulses in Figure 3a).

**Figure 4:**
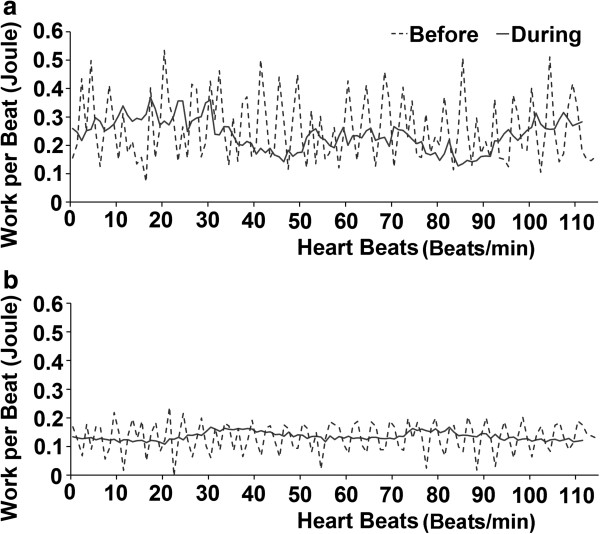
Variations in beat-to-beat working loads of the LV and LVAD before (a) and during (b) counter-pulsation control in the first experiment.

Table 3 shows the measurements of the working load and LVR values of the LV before and during counter-pulsation control in all experiments, and Figure 4 shows the beat-to-beat working load variations in the LV and LVAD during 110 successive blood pumping periods before and during counter-pulsation control in the first experiment. As shown in Table 3, the standard deviations of the LV load were reduced from 0.113 to 0.055 Joules/beat (51.3% reduction) in Case 1, from 0.106 to 0.034 Joules/beat (67.9% reduction) in Case 2, and from 0.123 to 0.035 Joules/beat (71.5% reduction) in Case 3 during counter-pulsation control. The LVR values were also reduced from 64.5% to 9.8% (84.8% reduction) in Case 1, from 60.0% to 10.4% (82.7% reduction) in Case 2, and from 73.7% to 8.7% (88.2% reduction) in Case 3 during counter-pulsation control. On the contrary, the working load of the LV was reduced in Cases 1 and 3, but was increased in Case 2 during counter-pulsation control.

**Table 3:** Variations in the working load and LVR values in the LV before and during counter-pulsation control

**Animal**	**Load of LV (Joules/beat)**		**LVR of LV (%)**		**LV load reduction (%)**
	**Before**	**During**	**Before**	**During**	
1	0.258 ± 0.113 (n = 115)	0.238 ± 0.055 (n = 113)	64.5 (n = 114)	9.8 (n = 112)	7.6
2	0.273 ± 0.106 (n = 130)	0.318 ± 0.034 (n = 130)	60.0 (n = 129)	10.4 (n = 129)	−16.5
3	0.216 ± 0.123 (n = 120)	0.148 ± 0.035 (n = 120)	73.7 (n = 119)	8.7 (n = 119)	31.5

Data are presented as means ± standard deviation format.

Table 4 shows the heart rate variations and occurrences of premature ventricle contractions (PVCs) before and during counter-pulsation control (each sample was of 5-min duration). Heart rate variations were reduced from 101.4 ± 13.6 to 90.8 ± 1.3 bpm in Case 1, from 80.5 ± 13.8 to 90.1 ± 1.0 bpm in Case 2, and from 102.8 ± 11.4 to 98.7 ± 1.2 bpm in Case 3 during counter-pulsation control. There were 3 to 12 PVCs before counter-pulsation control, but no PVCs were observed during counter-pulsation control.

**Table 4:** Heart rate variations and PVC occurrences before and during counter-pulsation control

**Animal**	**Heart rate**		**PVC**	
	**Before**	**During**	**Before**	**During**
1	101.4 ± 13.6	90.8 ± 1.3	7 (n = 115)	0 (n = 113)
2	80.5 ± 13.8	90.1 ± 1.0	3 (n = 130)	0 (n = 130)
3	102.8 ± 11.4	98.7 ± 1.2	12 (n = 120)	0 (n = 120)

Before counter-pulsation control, the working load of the LV in the diastole phase varied under 85% of the average working load of the LV (p-value < 0.05), but during counter-pulsation control, this working load variation in the diastole phase reduced to under 35% of the average working load (p-value < 0.05). In addition, most of the LVR values were over 4% before counter-pulsation control (p-value < 0.05) and were under 20% during counter-pulsation control (p-value < 0.05).

## Discussion

In this study, a counter-pulsation algorithm for a pneumatic LVAD was implemented, and the clinical effects of counter-pulsation control on the working load of the native heart were evaluated in three short-term animal experiments. In the animal experiments, the mean values of the working load of the LV were not markedly affected by counter-pulsation control (e.g., the working load of the LV decreased during counter-pulsation control in Cases 1 and 3, but increased during counter-pulsation control in Case 2). This occurred because the working load of the LV is affected by temporary variations in cardiac outflow and aortic pressure; therefore, if the cardiac outflow is temporarily increased, the working load of the native heart can increase even when the aortic pressure is actually reduced by counter-pulsation control. On the contrary, the deviations in the working load of the LV were markedly reduced during counter-pulsation control (51.3 – 71.5% reduction), as were LVR values (82.7 – 88.2% reduction). These results suggest that counter-pulsation control of the LVAD reduces the maximal deviation of the cardiac load and therefore also the occurrence of temporary severe cardiac load variations.

The occurrence of ventricular arrhythmia during VAD support is a topic of debate. For example, in studies by Moroney *et al*. [19] and Arai *et al*. [20], the frequency of the occurrence of ventricular arrhythmia during VAD support was reduced after VAD support, but ventricular arrhythmias during VAD support were temporary and converted spontaneously to normal pacing; the effect on patient survival was not significant. On the contrary, Harding *et al*. reported that LVAD placement induces changes in the contractile function of the failed native heart, and the early period after initiation of LVAD support of the failed native heart is associated with a relatively high incidence of significant ventricular arrhythmias after LVAD placement [21,22]. In addition, Brenyo *et al*. reported that the patient’s pre-LVAD history of ventricular arrhythmia is a significant predictor of the occurrence of post-LVAD ventricular arrhythmia, and the occurrence of post-LVAD ventricular arrhythmia significantly increases the risk of mortality, particularly within the first month [23]. Furthermore, to enhance the survival rate of LVAD patients, Refaat *et al*. [24] and Cantillon *et al*. [25] implanted a cardioverter-defibrillator with a LVAD, and Dandamudi *et al*. [26] performed catheter ablation to resolve ventricular tachycardia in VAD patients. Our findings demonstrate that LVAD counter-pulsation control can stabilize the blood pumping of the native heart and prevent the temporary severe cardiac load variations that may induce ventricular arrhythmias during LVAD support; e.g., the heart rate variations were reduced from 11.4 – 13.8 to 1.0 – 1.3 bpm during counter-pulsation control. In addition, PVCs were eliminated during counter-pulsation control. These experimental results demonstrate that the application of counter-pulsation control can seriously reduce the occurrence of abnormal heart beats during LVAD support without any additional surgical operations. Considering this result, it may be recommended to apply the pulsatile LVAD or, when the continuous LVAD was applied, control the device with pulsatile control profile (with counter-pulsation) [12] to the patients with high-risk of ventricular arrhythmias. However, considering the limitations of the current study (the experimental cases were very limited, the measurements were performed for only 3 h after beginning the LVAD operation, the animals were under anesthesia during the measurements, and the mechanism of ventricular arrhythmia during LVAD support is complicated), further long-term investigations of the clinical efficacy of LVAD counter-pulsation control are required [27].

In this study, the v-ECG signal was measured by attaching a unipolar electrode directly to the LV apex. This v-ECG signal is affected by changes in the local ion concentration at the LV apex and thus differs from the conventional ECG signal, which is affected by changes in the ion concentration of the whole heart tissue. However, abnormal depolarization of the local ventricular muscle occurs occasionally due to the unstable noise caused by the mechanical stress on the heart muscle. In these exceptional situations, counter-pulsation control based on the v-ECG signal can be temporarily unstable. However, the risk of this occasional instability in v-ECG-based counter-pulsation control may not be serious because the frequency of abnormal ventricular muscle depolarization is very low. Indeed, even when such an exceptional case occurs, the pacing of the LVAD returns to the normal counter-pulsation operation immediately following the next cardiac contraction. However, it is important to investigate possible clinical situations in which the v-ECG-based counter-pulsation control becomes unstable and to establish additional error-correction protocols to prevent device malfunction in such abnormal situations.

## Conclusions

Our findings suggest that counter-pulsation control of the pulsatile LVAD can reduce severe cardiac load variations and stabilize the blood pumping of the native heart. However, the average working load is not markedly affected by application of counter-pulsation control because it is also influenced by temporary cardiac outflow variations. Based on these results, we expect counter-pulsation control of the pulsatile LVAD to improve the long-term safety of patients with heart failure; however, more long-term survival cases or clinical studies are required to confirm our results.

## Abbreviations

LVAD: Left ventricular assist device; LV: Left ventricle; v-ECG: Ventricular electrocardiogram; ABP: Arterial blood pressure; LVR: Load variation rate; PVC: Premature ventricle contraction.

## Competing interests

The authors declare that they have no competing interests.

## Authors’ contributions

CSW Designed the LVAD, performed animal experiments (data acquisition and interpretation), investigated the experimental data (statistical process), and wrote the manuscript. NKW Suggested the overall concept of the paper, investigated the data (acquisition and interpretation), and wrote the manuscript. LKM, SEB, KIY Designed the LVAD, performed animal experiments (data acquisition), and investigated the data (statistical process). WYS, WHM, KHH, NMR, PSM Designed the animal experiments, performed animal experiments (surgical implantation) and investigated the data (interpretation). All authors read and approved the final manuscript.
